# Organizational and Occupational Stressors, Their Consequences and Coping Strategies: A Questionnaire Survey among Italian Patrol Police Officers

**DOI:** 10.3390/ijerph15010166

**Published:** 2018-01-21

**Authors:** Daniela Acquadro Maran, Massimo Zedda, Antonella Varetto

**Affiliations:** 1 Department of Psychology, Università di Torino, Via Verdi, 10, 10124 Torino, Italy; massimo@zedda.it; 2 Città della Salute e della Scienza, Corso Bramante, 88, 10126 Torino, Italy; avaretto@cittadellasalute.to.it

**Keywords:** anxiety, distress, health, workplace, police

## Abstract

**Background**: Traditionally, workers employed in police forces have been found to be exposed to a high risk of distress. Several studies reported that the main stressors were associated more with organizational aspects, whilst other researchers underlined that the main stressor were associated more with operational issues. The aim of this research was to investigate operational and organizational stressors, their consequences also in terms of anxiety and the coping strategies adopted. **Methods**: We compared Patrol Police Officers working in the Operational Service (Outdoor Patrol Officers) and those in the Interior Department (Indoor Patrol Officers) in the same Municipal Police force. **Results**: The results revealed that both Outdoor Patrol Officers and Interior Patrol Officers suffered from organizational and occupational stressor. Outdoor Patrol Officers appeared more willing to use different coping strategies, whereas Indoor Patrol Officers used avoidance strategies. This allows Outdoor Patrol Officers to explore new responses and approaches to deal with situations which—owing to the type of work—it is impossible to change. Outdoor Patrol Officers appeared better equipped to change their attitude to work than Indoor Patrol Officers. **Conclusion**: Interventions on both organizational and operational stressors would improve the quality of Patrol Police Officers’ working life and have positive repercussions on the service offered to the general public.

## 1. Introduction

Traditionally, workers employed in police forces have been found to be exposed to a high risk of distress [1]. Work-related disability and productivity loss as a result of distress and depression are critical determinants of quality of life and contribute significantly to health burden [2] as well as indirect medical costs [3]. The risk of distress derives from their operational duties (such as patrol activities, traffic control, criminal investigations, crime prevention, community services) and organizational tasks (e.g., selection and management of personnel/human resources, training of new recruits, public information, record keeping, bureaucratic procedures) [4]. Operational stressors are defined as any persistent psychological difficulty resulting from operational duties performed as part of the job. They are inherent in the nature of the job, which implies the use of force, making decisions in critical situations, risks to own safety and that of colleagues, attending the scenes of accidents and injuries (sometimes death), exposure to suffering and violence [5]. The organizational side also has sources of distress: inactivity, boredom, bureaucratic administration, relationships with colleagues and superiors, the public’s perception of police work and inadequate relationships with supervisors are some examples of the stressors that have been recognized in research involving police officers [6]. In literature, about the source of stressors in this population there is a controversy: the nature of main stressors is organizational or operational? In their study on Indian police personnel, Suresh et al. [7] reported that the main stressors were associated more with organizational aspects (never being off duty, lack of time to spend with family, political pressure from outside the police department, inadequate salary/facilities) than with operational issues (e.g., having to cope with the injured/dead, exposure to suffering, exhumation). It is interesting to note that there appear to be no differences between armed and unarmed police officers: in a study conducted by Prasad [8], both groups of officers had high scores on items measuring organizational stressors. Garbarino, et al. [9] investigated conditions of distress in people with symptoms of depression, anxiety and burn-out: their results suggest that organizational stressors (less autonomy in making decisions, poor relationships between colleagues and superiors, less opportunity of reward, demanding work environment and high commitment) might be associated with higher levels of depressive symptoms.

Other researchers, e.g., Leino [10], focused their attention on operational stressors. In a study among Finnish patrol police officers, the author reported that operational stressors (e.g., injuries caused by physical violence) worsen psychological health, increasing the risk of distress. The study conducted by Leino, et al. [11] revealed that threats or assaults with deadly weapons had an independent association with distress. In an Italian investigation, Setti and Argentero [12] compared the organizational and operational stressors. Their findings shown that the operational stressor score was slightly higher than the organizational stressor score. 

Ways of coping with organizational and operational stressors and their effectiveness have been evaluated with reference to the coping strategies used [13]. This study reported that police officers cope with the distress caused by operational stressors by using their experience and also by adopting strategies that could be useful to decrease the perceived distress, such as active coping, social support and positive reinterpretation of the situation. The same study also pointed to a possible relationship between operational activities and some strategies that could increase the perceived distress, e.g., excessive display of emotions, escape and psychological detachment from stressful events [14,15,16]; the consequences are usually depression and physical symptoms [17,18]. 

The aim of this research was to investigate the nature of stressors (operational and organizational) in Italian Patrol Police Officers, their consequences also in terms of anxiety and the coping strategies adopted. The originality of this study lies in the fact that, to the best of our knowledge, this is the first time a comparison has been made among Patrol Police Officers working in two different sectors but in the same police force. This police force is the Municipal Police, located in a large city in the North of Italy. The participants in this study were Patrol Police Officers, the lowest rank of officers in the organizational structure. Officers in this police force operate within the community; they are directly responsible for their actions and undertake a range of tasks depending on the needs of the community (e.g., maintenance of public order, law enforcement, authorizations, permits, etc.) [19]. There are other hierarchical positions within the organization: The Chief of Police, Executives, Unit Managers, Officers, Non-Commissioned Officers. As reported in previous investigations involving this population, Patrol Police Officers are exposed to a high risk of distress, which appears to be due to their low-ranking position [20]. As in all major Italian cities, in this Municipal Police Force Patrol Police Officers work shifts so that their services are provided 24 h a day, 7 days a week. This police force was established more than 200 years ago in Italy and there are 10.4 police officers for every 10,000 inhabitants. Women account for 25.7% of the workforce. In the city where the study was conducted this police force has 1840 officers, 1018 of whom are patrol police officers (55.3%). The number of men and women is roughly equal (54.5% men), with an average age of 45.6 years and an average of service of 15.3 years.

The city’s urban environment has undergone some profound changes as a result of transformations in the typical type of work performed (once mainly centred around the metallurgy industry) and migratory flows, that have altered the characteristics of social disadvantage, interpersonal relationships, high-risk areas and service offered. The distinguishing feature of this police force is its mission, which is closely linked to the community and involves a variety of activities: prevention, control and information. Patrol Police Officers work in two different sectors of intervention, namely:

- Operational Service: responsible for enforcing the law, intervening directly in cases of assault (e.g., domestic violence), investigating crimes, ensuring safety (on the roads, in public places);
- Interior Department: e.g., responsible for relations with the public, information and crime-prevention projects, personnel management.

Thus, Patrol Police Officers working in the Operational Service are involved in action on the ground, they face the sufferance, the delinquency and the economic difficulties by the population. The Patrol Police Officers working in the Interior Department have back office duties that involves both the organization (such as the timetable and shift, organization of training) and the population (information office, payment of fines and so on). To facilitate the presentation in this paper, we have labelled Patrol Police Officers working in the Operational Service as Outdoor Patrol Officers and Patrol Police Officers working in the Interior Department as Indoor Patrol Officers.

Due to the nature of their work, our hypothesis is that the Outdoor Patrol Officers suffer of occupational stressors more than Indoor Patrol Officers, while Indoor Patrol Officers of organizational stressors more than Outdoor Patrol Officers (Hypothesis 1). Moreover, we expected that Outdoor Patrol Officers experienced higher level of work-related distress that affect their quality of life, more than Indoor Patrol Officers (Hypothesis 2). Finally, that the Outdoor Patrol Officers are prone to use, more than Indoor Patrol Officers, coping strategies oriented to excessive display of emotions, escape and psychological detachment from organizational and occupational stressful events (Hypothesis 3).

## 2. Method

We conducted a survey to measure the level of operational and organizational distress experienced by Outdoor Patrol Officers and Indoor Patrol Officers, the consequences and the coping strategies adopted. We chose the quantitative method because this assesses the effects of exposure to distress and how participants respond. We analysed the data obtained to report means and determine the interactions of the variables, namely the type of duties involved in the job. Thus, this research can be replicated due to its ability to investigate the connection between variables through closed-end questions, use of structured approaches and use of statistical procedures [21].

### 2.1. Ethical Statement

The study presented in this article conformed to the provisions of the Declaration of Helsinki in 1995, revised in Edinburgh 2000 (World Medical Association, 2001). All ethical guidelines were followed, as required for conducting human research, including adherence to the legal requirements of Italy. The research project was approved by a committee, composed of two unit managers (one with a degree in law, the other a clinical psychologist) and one supervisor (head of general affairs). Since there was no medical treatment or other procedures that could cause psychological or social discomfort to participants, additional ethical approval was not required. With the approval of the managers, participants were asked for authorization to administer the questionnaire. The cover sheet clearly explained the research aim, the voluntary nature of participation, the anonymity of the data and the elaboration of the findings. Thus, returning the questionnaires implied consent. Participants volunteered in the research without receiving any reward.

### 2.2. Participants

The respondents were 531 (52.16%), the sector of employment was not indicated by 51 patrol officers (they were excluded from analysis). The participants were 266 Outdoor Patrol Officers and 214 Indoor Patrol Officers. The Outdoor Patrol Officers were the 41.8% of the total number of patrol officers in the Operational Service. They comprised 143 males and 123 females, with an average age of 40.45 years (SD 6.80, range 25–59). The largest proportion were married (107, 40.2%), 22 (8.3%) were separated and 55 (20.7%) were cohabiting/engaged. The majority of respondents (242, 91%) had one or more children. The Indoor Patrol Officers were the 57.7% of the total number of patrol officers in the Interior Department and comprised 110 males and 104 females. Their average age was 41.85 (SD 9.45, range 21–65). The largest proportion were married (97, 45.3%), 13 (6.1%) were separated and 28 (13.1%) were cohabiting/engaged. Most part of respondents (172, 80.4%) had one or more children.

### 2.3. Materials

The self–administered questionnaire was composed of different sections: the first part contained the introduction and the purpose of the research, the instructions for filling out the questionnaire, the anonymity and privacy statements. This was followed by specific questionnaires to examine the work-related stressors specific to Police Officers (Police Stress Questionnaire) and levels of distress perceived in other areas of life (Distress Thermometer), a questionnaire to measure anxiety (State Trait Anxiety Inventory) and finally a questionnaire to identify coping strategies (Brief Cope). The last page contained socio-personal data (gender, age, marital status, household composition) and working sector.

The Police Stress Questionnaire [22] consists of two scales (each made up of 20 items) designed to assess operational (PSQ-Op) and organizational (PSQ-Org) stressors. The PSQ-Op measures distress related, for instance, to ‘shift work’, ‘working alone at night’, ‘over-time demands’, ‘risk of injury on the job.’ The PSQ-Org measures distress associated, for instance, with ‘dealing with co-workers’, ‘the feeling that different rules apply to different people’, ‘feeling like you always have to prove yourself to the organization’. Participants rate each item on the scale according to the level of distress it has caused recently using a 7-point scale that ranges from ‘no stress at all’ to ‘a lot of stress’. The Italian version of the questionnaire was developed by Setti and Argentero [23]. In the present study, Cronbach’s alpha was respectively 0.92 and 0.94.

The Distress Thermometer is a tool developed by Roth and colleagues [24], originally to measure distress in cancer patients and which has recently also been used to measure distress in association with anxiety and depression e.g., in workers after a traumatic incident [25]. The Italian version by Grassi et al. [26] has already been used to assess distress in police officers [27]. It is a rapid screening instrument in which participants rate their level of distress during the previous week on a visual analogue scale that ranges from 0 (not distressed) to 10 (extremely distressed). As indicated by the National Cancer Institute (NCI), most studies use a cut-off score of 4 or 5. The Distress Thermometer also covers questions about the sources of distress classified as practical problems (5 items, e.g., ‘childcare’), family problems (3 items, e.g., ‘dealing with partner’), emotional problems (6 items, e.g., ‘sadness’), spiritual/religious concerns (1 item). The scale also investigates the physical problems (21 items, e.g., ‘nose dry/congested’). It consists of 37 items. In this study, internal consistency was Cronbach’s alpha = 0.93.

The STAI Y1 and Y2 scales [28] rate state and trait anxiety. The Y1 form measures the person’s current anxiety about an event, that is the temporary interruption in the emotional continuum characterized by a subjective feeling of tension and associated with the arousal of the autonomic nervous system. The Y2 form measures how an individual usually feels in typical daily situations experienced by everyone, revealing the anxiety level as a personality trait. Each of the two scales comprises 20 items rated on a 4-point scale. Total scores can range between 20 and 80, with 40 as the threshold value predictive of anxiety symptoms. A rating scale can also be used to define the level of severity: from 40 to 50 mild, 50 to 60 moderate, >60 severe. In this study, the Italian version was used [29]. Construct validity was examined by computing the internal consistency of the two subscales. The Cronbach’s alpha in this study was respectively = 0.85 and 0.84.

The Brief COPE [30] is a self-administered questionnaire used to assess 14 coping strategies: Self-distraction, Active coping, Denial, Substance use, Emotional support, Instrumental support, Behavioral disengagement, Venting, Positive reframing, Planning, Humour, Acceptance, Religion, Self-blame. It rates 28 coping responses under stressful conditions, with scores ranging from1 (I would not normally do this) to 4 (I would usually do this). In this study, the Italian version was used [31] (Cronbach’s alpha = 0.80).

Statistical analyses were performed using the statistical software SPSS, version 20. Descriptive measures (means ± SD) were calculated for all test variables for all groups of participants. The *t*-test was performed to evaluate the statistical significance of the observed differences (*p* < 0.05 was considered to be significantly different). Correlation were calculated to examine the relationship between organizational and operational stressors, perceived stress, anxiety scores and coping strategies. Multiple linear regression analysis was performed with the subject’s perception of distress as the dependent variable with PSQ-Op, PSQ-Org, STAI Y1-Y2 and coping strategies as explanatory variables.

### 2.4. Procedure

The questionnaires were distributed to 1010 Patrol Police Officers (response rate 47.5%). After obtaining the permission of the managers, we planned the most suitable days for presenting the study and distributing the questionnaires to the Patrol Police Officers. Data were collected in 3–21 February 2014. Managers gave us some suggestions about distribution of the questionnaire: (1) to distribute the questionnaires during the shift change-over time, as this was the time when the majority of Patrol Police Officers were present and (2) to leave additional copies at the headquarters for those who were not present because they were either on vacation or on sick leave. The participants were asked to fill out all parts of the questionnaire and post it into a sealed box with a slot in the top, which was left in the changing room. This procedure had already been adopted in previous studies [27] and enabled us to guarantee privacy and anonymity. On the box, there was a label indicating the title of the study, the expected collection date and the contact details of two of the authors of this paper for anyone who required more information (e.g., on how to fill out the questionnaire, the aim of the research, the methods that would be used to process their data). None of the participants used this opportunity. The questionnaires were collected 15 days later for all participants.

## 3. Results

### 3.1. Descriptive Statistics

The Patrol Police Officers’ scores on the PSQ-Op and PSQ-Org scales did not generally reveal high levels of distress (none of the responders rated their distress as more than 5 on a scale from 1 to 7). In Distress Thermometer, Outdoor Patrol Officers and Indoor Patrol Officers obtained similar scores that did not reach the cut-off level of 4, which was considered the threshold for distress (see Table 1).

The mean scores of STAI Y1 and Y2 revealed that Outdoor Patrol Officers reached the cut-off level for moderate anxiety on both the trait and state scales, while Indoor Patrol Officers reached the cut-off level for mild anxiety on both the trait and state scales. 

Considering the mean scores, overall findings showed that Active Coping, Acceptance and Planning were the most common strategies used by all Patrol Police Officers. There were significant differences in anxiety (state and trait) and coping strategies scores. Outdoor Patrol Officers experienced, more than their colleagues Indoor Patrol Officers, anxiety symptoms (both trait and state anxiety). Indoor Patrol Officers were more prone to use Self-distraction, Denial and Religion, while Outdoor Patrol Officers were more prone to use Substance use and Instrumental Support. 

### 3.2. Inferential Statistics

A correlation matrix examined the relationships between scores on the PSQ-Op, PSQ-Org, Distress Thermometer and its problems, STAI Y1 and Y2, Brief Cope scales for both groups of Police Patrol Officers (see Table 2).

About operational stressors, Outdoor Patrol Officers’ PSQ-Op and PSQ-Org scores were significantly related. PSQ-Op was negatively related to STAI Y2 and significantly related to the Distress Thermometer score. Moreover, among problems PSQ-Op was negatively related to Practical (*r* = −0.43, *p* = 0.000), Relational (*r* = −0.29, *p* = 0.000), Emotional (*r* = −0.48, *p* = 0.000) and Physical problems (*r* = −0.53, *p* = 0.000). Among coping strategies, PSQ-Op was related to Behavioral disengagement, Self-distraction, Substance use, Self-blame, Venting, Instrumental support and Emotional support. Outdoor Patrol Officers’ PSQ-Org scores were significantly related to the Distress Thermometer score and to the STAI Y2. Among the items on the Distress Thermometer, PSQ-Org negatively related to Practical (*r* = −0.38, *p* = 0.000), Relational (*r* = −0.24, *p* = 0.000), Emotional (*r* = −0.44, *p* = 0.000) and Physical problems (*r* = −0.42, *p* = 0.000). PSQ-Org was significantly related to some coping strategies: Denial, Self-distraction, Behavioral disengagement, Substance use, Self-blame, Instrumental Support, Venting, Emotive support. 

Turning to Indoor Patrol Officers, PSQ-Op and PSQ-Org scores were significantly related. PSQ-Op was significantly related to the Distress Thermometer score and negatively to some problems: Practical (*r* = −0.19, *p* = 0.007), Relational (*r* = −0.24, *p* = 0.001), Emotional (*r* = −0.26, *p* = 0.000) and Physical (*r* = −0.30, *p* = 0.000). Among coping strategies, PSQ-Op was significantly related to the following coping strategies: Denial, Self-distraction, Behavioral disengagement and Self-blame. Indoor Patrol Officers’ PSQ-Org scores were significantly related to the Distress Thermometer score and negatively to some problems: Practical (*r* = −0.26, *p* = 0.000), Relational (*r* = −0.16, *p* = 0.017), Emotional (*r* = −0.29, *p* = 0.000) and Physical (*r* = −0.34, *p* = 0.000). PSQ-Org was significantly related to STAI Y1 and STAI Y2. Among coping strategies, PSQ-Org was significantly related to Self-distraction, Denial and Self-blame coping strategies. PSQ-Org was also negatively related to Substance use.

Multiple linear regression analysis showed that perceived distress in Operational Patrol Officers was correlated with PSQ-Op (*p* = 0.001), PSQ-Org (*p* = 0.002) and STAI-Y1 (*p* = 0.001) and negatively correlated with Planning (*p* = 0.044) and Humour (*p* = 0.009) (R2 = 0.49, F = 6.98, *p* = 0.000). In Indoor Patrol Officers, multiple linear regression analysis showed that perceived distress was correlated with PSQ-Op (*p* = 0.025), STAI-Y1 (*p* = 0.042) and negatively correlated with Active Coping strategy (*p* = 0.037) (R2 = 0.26, F = 2.14, *p* = 0.018). The analysis found no correlations with other independent variables.

## 4. Discussion

In our study findings shown that Outdoor Patrol Officers were more vulnerable to both sources of distress than Indoor Patrol Officers and not primarily to occupational stressors, thus our Hypothesis 1 was not confirmed. As already observed by Leino [10] and Leino et al. [11], such stressors determine higher levels of anxiety in these Officers, more than in Indoor Patrol Officers. In line with the findings of studies on prolonged exposure to critical events, Outdoor Patrol Officers reported suffering from anxiety more than Indoor Patrol Officers, confirming the Hypothesis 2. As found by Paton [32], such feelings affect how they perceive the effectiveness of their actions.

Moreover, Outdoor Patrol Officers used coping strategies activated in some cases by an internal cause (e.g., instrumental support involves ask for help, sharing the problem) and in others by an external one (e.g., substance use to help them deal with stressful situations). The use of some strategies, such Planning and Humour, mitigate the perceived distress. Indoor Patrol Officers made more use of strategies activated by an internal locus (e.g., emotional support). When they use the Active Coping strategy, the perceived distress decrease. They also appeared to have a greater desire to control their anxiety and emotional distress, although such control resulted in somatization. As observed by Pietrantoni et al. [15], avoidance and detachment from stressful events could determine severe depression and physical problems. 

Anxiety, caused by distress, could affects in Outdoor Patrol Officers the perceptions of efficiency in relation to the task performed within the organization: the risk is the emotional exhaustion [23,33]. Indoor Patrol Officers use self-distraction, negation and self-blame strategies to cope with increased operational distress. Such strategies are used by people who tend to avoid damage, move away from the problem and stem from a feeling of being unable to handle the stressful situation [34]. The strategies used imply self-blame (for being unable to cope adequately with the problem) but also a refusal to acknowledge the problem (thus reducing the sense of responsibility). Outdoor Patrol Officers also adopted avoidance strategies but appeared to handle stressful situations more effectively: they used a wider variety of methods of adaptation than Indoor Patrol Officers. The same problems and the same strategies emerged from the responses to organizational stressors: Outdoor Patrol Officers adopted more varied strategies, Indoor Patrol Officers used avoidance strategies. Outdoor Patrol Officers are more prone to explore new responses and approaches (novelty seeking) to deal with situations which—owing to the type of work—it is impossible to change. The Hypothesis 3 was partially confirmed: Outdoor Patrol Officers appeared better equipped to change their attitude to work but they do not tend to use coping strategies oriented to excessive display of emotions. At the same time, they are prone to use escape and psychological detachment from stressful events. In these subjects, there was a relation between the increase in organizational stressors and the Behavioral disengagement strategy, which did not emerge in relation with operational stressors. Moreover, the attribution of the cause of distress (operational, organizational) coincides with a reduced effort to deal with the stressor in general life. The feeling associated with this strategy is one of helplessness [35] and ruminations [36]. Indoor Patrol officers might be more inclined to credit external factors in success than in failure and ascribed greater internal control in failure than in success due to higher level of self-blame [37]. Outdoor Patrol Officers do not think there is anything they can do to improve the situation, because it does not depend on them but on an external factor (the organization). This work sheds light on the importance of conducting a combined study to investigate both operational and organizational stressors and their consequences that can negatively affect the physical and psychological health of Patrol Police Officers. As the best of our knowledge, this is the first time that in an Italian context a comparison of source of distress (operational, organizational) among different sector of intervention (Outdoor, Indoor) has been made. Findings shown that Outdoor Police Patrol Officers were more vulnerable than Indoor Patrol Officers to both sources of distress but appeared to handle stressful situations more effectively. As Pietrantoni and colleagues [15] underlined, this profession is characterized by stressful situations for which there is no solution: those workers are routinely exposed to distress thus, they are at risk of chronic disease [38]. However, it is possible to find strategies to limit the stressors and their consequences [39]. Understanding the sources of distress in this different sector of intervention (Outdoor, Indoor) could be useful in order to: (a) tailor training courses to suit the real needs of Patrol Police Officers, (b) increase their ability to cope with distress, (c) prevent the consequences of distress in terms of physical and psychological problems, (d) intervene before distress becomes chronic over their working rules, (e) improving symptom management ability and problem-focused coping skills to raise their life quality [40] and (f) online cognitive behaviour therapy to ensure privacy and cost-effectiveness for police officers [41]. People who are more able to adapt and have more active coping strategies have greater control over their working rules. They are therefore more likely to improve the way they handle critical situations and this, in turn, can make it easier for them to adapt to stressors. Conversely, higher anxiety levels make it more difficult to find suitable solutions for coping with distress. 

Some limits of this research should be underlined. First, the sample we investigated was self-selected, in that the people we contacted were free to decide whether or not to take part. The sample may not have been particularly representative of the universe of reference. The results must therefore be considered as restricted to the participants in the study. The lack of information about non-respondents inevitably limits our conclusions by preventing any further analysis of responder bias. Second, we did not consider variables such as gender and experience. These could have an impact on resilience, the ability to cope with problems associated with professional role and identity [42]. Third, stress affects the immunity and causes chronic medical diseases including coronary artery disease [43]. This study did not report the prevalence of chronic medical diseases which is associated with higher level of stress, poor subjective health and impaired functional status [44]. Furthermore, we did not consider the possible impact of neighbourhood factors on distress levels. A patrol officer in a neighbourhood with high levels of violence and drug activity may experience or report higher stress levels than an officer in a quiet neighbourhood with few social problems. Additional research should be conducted with the support of more sophisticated measures to examine whether certain variables (such as gender, experience, neighbourhood), have an impact on the perception of distress, its consequences, the coping strategies used and resilience. Moreover, this study would be more interesting and scientific if we could have the opportunity of clinical assessments for the police patrol officers with positive test results in depression and anxiety symptoms. Unfortunately, because of the constraints (time, organizational and financial obstacles) clinical evaluations have not been made. Further studies to complete these assessments (also with longitudinal nature) are needed.

## 5. Conclusions

In conclusion, this study provides some indications for municipal police forces when planning preventive actions and measures for Patrol Police Officers. In general, this population should be supported by specific training to reduce the risk of develop chronic diseases. As described by Setti and Argentero [23], to improve the ability to cope with distress (operational, organizational), regular psychological support, group intervention, structured training program on sources of distress (also the life-events), could be effective. 

Moreover, our findings might be helpful to gain a better insight into the most effective methods of intervention on the basis of the specific characteristics of both groups. For Outdoor Patrol Officers, attention should be placed on maintaining their ability to use a broad range of coping strategies (in particular Planning and Humour), while increasing their use of it. In general, some strategies (especially behavioural disengagement, linked to the increase in organizational stressors), need to be monitored, as these can lead to disillusionment and a greater risk of chronic stress [32]. For subjects at risk of chronic stress, it might be useful, for example, to employ behavioural techniques to cope with demanding situations and manage distress (e.g., autogenic training, relaxation techniques). As regards Indoor Patrol Officers, attention needs to be paid to increasing and improving their use of coping strategies, to limit the stressors and their consequences. These subjects could benefit from discussion groups—e.g., focus groups, support groups—on topics such as re-evaluating their work-related motivations. This type of intervention could be directed at exploring an overly negative view of their work (e.g., their contribution to long-term institutional goals), while also scheduling activities likely to underline the importance and meaning of their role (e.g., highlighting the importance of the work of Indoor Patrol Officers in strategic objectives). Interventions on organizational stressors might be helpful to improve the wellbeing of all police patrol officers. For example, providing opportunities for them to talk about their malaise, promoting greater transparency in decision-making processes and personnel management. Interventions on organizational and operational stressors would improve the quality of Patrol Police Officers’ working life and have positive repercussions on the service offered to the general public. 

## Figures and Tables

**Table 1:** Means, Standard Deviation, Standard Error and Confidence Interval for Scores on the PSQ-Op and PSQ-Org, Distress Thermometer, STAI Y1 and Y2 forms and Brief COPE.

Measure	Outdoor Patrol Officers			Indoor Patrol Officers				
	M(SD)	SE	95% Cl(LL–UL)	M(SD)	SE	95% Cl(LL–UL)	*T*	*p*
PSQ-Op	3.06(1.22)	0.07	2.88–3.20	2.68(1.05)	0.07	2.32–2.81	3.554	0.380
PSQ-Org	3.42(1.33)	0.08	3.24–3.60	3.06(1.25)	0.09	2.55–3.15	3.045	0.360
Distress Thermometer	3.95(2.71)	0.19	3.15–4.85	3.99(2.52)	0.19	2.99–4.70	−0.143	0.304
Practical Problems	7.06(1.03)	0.06	6.91–7.17	7.35(5.50.81)	0.06	7.24–7.47	−1.548	0.122
Family Problems	5.50(0.68)	0.04	5.41–5.58	5.59(0.69)	0.05	5.51–5.70	−1.368	0.411
Emotional Problems	10.39(1.51)	0.09	10.16–10.54	10.57(1.43)	0.10	10.40–10.81	−1.198	0.232
Spiritual/religious concerns	1.93(0.25)	0.02	1.89–1.96	1.97(0.18)	0.01	1.94–1.99	−0.994	0.321
Physical Problems	38.77(2.96)	0.18	38.39–39.14	39.48(2.87)	0.20	39.16–39.96	−1.064	0.288
STAI-Y1	56.47(4.29)	0.26	55.85–56.99	47.00(11.73)	0.80	42.88–51.82	11.201	0.000
STAI-Y2	55.61(4.34)	0.27	55.03–56.17	47.01(11.18)	0.77	44.78–52.61	10.583	0.000
Brief COPE								
Self-distraction	1.94(0.81)	0.05	1.81–2.00	2.02(0.78)	0.08	1.98–2.16	−0.844	0.009
Active Coping	3.30(0.71)	0.04	3.17–3.37	3.27(0.53)	0.07	3.16–3.46	0.427	0.320
Denial	1.32(0.58)	0.03	1.23–1.38	1.39(0.32)	0.05	1.25–1.49	−1.149	0.025
Substance use	1.05(0.25)	0.01	1.01–1.08	1.03(0.02)	0.01	0.99–1.05	0.813	0.042
Emotional support	2.01(0.79)	0.05	1.91–2.11	2.09(0.61)	0.07	1.85–2.19	−0.956	0.339
Instrumental support	2.43(.79)	0.05	2.31–2.52	2.39(0.60)	0.07	2.30–2.60	0.550	0.049
Behavioral disengage	1.40(0.61)	0.04	1.31–1.45	1.41(0.37)	0.06	1.34–1.44	−0.189	0.850
Venting	2.39(0.74)	0.05	2.28–2.47	2.26(0.55)	0.07	2.10–2.41	1.539	0.125
Positive reframing	2.74(0.80)	0.05	2.59–2.80	2.64(0.69)	0.08	2.44–2.83	1.037	0.300
Planning	3.34(0.74)	0.04	3.23–3.43	3.22(0.50)	0.07	3.08–3.39	1.453	0.147
Humour	2.19(0.77)	0.05	2.09–2.29	2.24(0.65)	0.07	2.01–2.35	−0.579	0.563
Acceptance	3.03(0.76)	0.05	2.94–3.14	3.08(0.58)	0.07	2.93–3.23	−0.612	0.541
Religion	1.82(0.95)	0.06	1.66–1.90	1.88(0.93)	0.09	1.70–1.90	−0.528	0.598
Self-blame	2.77(0.70)	0.04	2.70–2.88	2.84(0.55)	0.07	2.27–3.03	−0.864	0.038

Note. PSQ-Op = Operational stressor; PSQ-Org = Organizational stressor; STAI-Y1 = state anxiety; STAI-Y2 = trait anxiety; M = Means; SD = Standard deviation; SE = Standard error; Cl = confidence interval; LL= lower limit; UL = upper limit; GDL for the *t*-tests are 265.

**Table 2:** Correlation among PSQ-Op and PSQ-Org, Distress Thermometer, STAI Y1 and Y2 forms and Brief COPE.

	1	2	3	4	5	6	7	8	9	10	11	12	13	14	15	16	17	18	19
1. PSQ-Op	-	0.75 **	0.35 **	0.03	−0.04	0.31 *	−0.01	0.34 **	−0.04	0.09	0.02	0.22 *	−0.06	−0.02	−0.10	0.12	−0.09	0.11	0.25 *
2. PSQ-Org	0.74 **	-	0.31 **	−0.07	−0.10	0.32 *	0.05	0.36 **	−0.21 *	0.13	0.05	0.14	0.00	0.06	−0.02	0.18	0.13	0.10	0.25 *
3. DT	0.52 **	0.50 **	-	0.17 **	0.13	0.13	−0.03	0.31 *	−0.15	0.10	0.01	0.21	−0.15	−0.05	−0.24 **	−0.09	0.03	0.07	0.08
4. STAI-Y1	0.03	−0.07	0.13	-	0.89 **	0.06	−0.10	0.05	0.02	−0.08	−0.01	0.05	−0.02	−0.16	−0.07	−0.10	−0.12	0.02	0.16
5. STAI-Y2	−0.17 **	−0.16 **	−0.11	0.39 **	-	−0.10	0.03	−0.25 *	0.06	0.11	0.21 *	−0.13	−0.08	−0.04	0.05	−0.04	0.00	−0.10	0.05
6. SD	0.21 *	0.20 *	0.23 *	−0.09 *	−0.16 *	-	0.07	−0.11	−0.09	0.23 *	0.38 **	0.07	0.12	0.10	0.12	0.03	−0.09	−0.02	0.24 *
7. AC	0.09	0.01	0.06	0.06	−0.15	0.12	-	−0.03	−0.28 *	0.13	0.09	−0.42 **	0.07	0.04	0.37 **	−0.05	0.20 *	−0.00	0.07
8. De	0.11	0.13 *	0.07	−0.23 **	−0.17 **	0.21 *	0.06	-	0.01	0.07	0.07	0.24 *	−0.11	0.05	−0.03	0.03	0.00	0.19 *	0.15
9. SU	0.18 *	0.14 *	−0.07	−0.04	−0.08	−0.06	−0.10	0.09	-	0.02	−0.05	0.34 **	−0.09	−0.07	−0.21 *	−0.06	0.06	−0.04	−0.13
10. ES	0.26 **	0.24 **	0.19 *	−0.06	−0.22 **	0.13 *	−0.17 *	0.15 *	0.02	-	0.60 **	0.08	0.34 **	0.14	−0.09	−0.01	0.00	0.00	0.14
11. IS	0.18 *	0.14 *	0.08	−0.08	−0.20 **	0.11	0.19 *	0.09	0.05	0.55 **	-	−0.01	0.38 *	0.21 *	−0.03	0.16	0.22 *	0.20 *	0.21 *
12. BD	0.12 *	0.14 *	0.11	−0.13 *	−0.14 *	0.20 *	−0.11	0.19 *	0.13 *	0.04	0.08	-	0.07	−0.06	−0.38 **	0.05	−0.03	0.04	0.15
13. Ve	0.26 **	0.27 **	0.18 *	−0.05	−0.23 **	−0.07	0.15 *	0.24 **	0.07	0.34 *	0.24 **	0.02	-	0.14	0.10	0.30 **	0.23 *	0.10	0.24 *
14. PR	−0.03	−0.01	−0.08	−0.01	−0.06	0.09	0.33 **	0.08	0.01	0.14 *	0.15 *	−0.14 *	0.13 *	-	0.23 *	0.44 **	0.35 **	0.22 *	0.12
15. Pl	−0.02	−0.10	−0.11	0.14 *	−0.06	−0.06	0.41 **	−0.12	−0.04	0.07	0.15 **	−0.20 **	0.23 **	0.42 **	-	0.15	0.21 *	−0.05	0.19 *
16. Hu	0.01	0.05	−0.15 *	−0.17 **	−0.07	0.08	0.08	0.17 *	0.15 *	0.11	0.10	0.06	0.17 *	0.37 **	0.06	-	0.29 *	0.18	0.17
17. Ac	−0.05	−0.10	−0.08	0.02	−0.02	−0.02	0.24 **	−0.02	0.03	−0.07	0.07	−0.03	0.24 **	0.34 **	0.40 **	0.29 **	-	0.31 *	0.14
18. Re	0.04	0.00	0.03	−0.15	−0.29 **	0.09	0.10	0.14	0.06	0.11	0.15 *	0.20 *	0.01	0.22 **	−14 *	0.10	0.14 *	-	0.22 *
19. SB	0.22 **	0.15 *	0.10	0.03	−0.14 *	−0.20 *	0.31 **	0.13 *	−0.04	0.27	0.22 *	0.05	0.30 *	0.18 *	0.29 *	0.15 *	0.16 *	0.14 *	-

Note. Correlations for Indoor Patrol Officers are presented above the diagonal, Outdoor Patrol Officers are presented below the diagonal. ** *p* < 0.001; * *p* < 0.05. PSQ-Op = Operational stressor; PSQ-Org = Organizational stressor; STAI-Y1 = state anxiety; STAI-Y2 = trait anxiety; DT = Distress Thermometer; SD = Self-distraction coping strategy; AC = Active coping strategy; De = Denial coping strategy; SU = Substance use coping strategy; ES = Emotional support coping strategy; IS = Instrumental support coping strategy; BD = Behavioral disengagement coping strategy; Ve = Venting coping strategy; PR = Positive reframing coping strategy; Pl = Planning coping strategy; Hu = Humour coping strategy; Ac = Acceptance coping strategy; Re = Religion coping strategy; SB = Self-blame coping strategy.
